# Coronary Computed Tomography Angiography for the Assessment of Sirolimus-Eluting Resorbable Magnesium Scaffold

**DOI:** 10.3390/life12101612

**Published:** 2022-10-15

**Authors:** Elisabetta Tonet, Alberto Cossu, Graziella Pompei, Rossella Ruggiero, Serena Caglioni, Daniela Mele, Alberto Boccadoro, Marco Micillo, Marta Cocco, Martina De Raffele, Melchiore Giganti, Simone Biscaglia, Fabio Sgura, Gerlando Preti, Youbing Yin, Yi Wang, Giorgio Quadri, Enrico Cerrato, Gianluca Campo

**Affiliations:** 1 Cardiovascular Institute, Azienda Ospedaliero-Universitaria di Ferrara, 44124 Ferrara, Italy; 2 University Radiology Unit, University of Ferrara, 44124 Ferrara, Italy; 3 Cardiovascular Department, Infermi Hospital, 47921 Rimini, Italy; 4 Cardiology Unit, Azienda Ospedaliero Universitaria di Modena, 41100 Modena, Italy; 5 Interventional Cardiology Unit, Ospedale di Conegliano, 31015 Conegliano, Italy; 6 Keya Medical, Seattle, WA 98104, USA; 7 Interventional Cardiology Unit, ASL TO3, Rivoli Hospital, 10094 Turin, Italy; 8 Interventional Cardiology Unit, ASL TO3, San Luigi Gonzaga University Hospital, 10094 Turin, Italy

**Keywords:** coronary computed tomography angiography, noninvasive fractional flow reserve, resorbable magnesium scaffold, percutaneous coronary intervention

## Abstract

Background: Little evidence to date has described the feasibility and diagnostic accuracy of coronary computed tomography angiography (CCTA) with noninvasive fractional flow reserve (CT-FFR) in coronary vessels with resorbable magnesium scaffold (RMS). Methods: The SHERPA-MAGIC is a prospective study enrolling patients receiving RMS. The present analysis considered patients undergoing CCTA 18 months after the index procedure. CCTA images were employed to investigate reabsorption status, luminal measurements, and noninvasive FFR. Three-year follow-up was available for all patients. Results: Overall, 26 patients with a total of 29 coronary arteries treated with 35 RMS were considered. The most frequently involved vessel was left anterior descendent (LAD). Median stent length was 25 (20–25) mm, with a median diameter of 3 (3–3.5) mm. At 18-month CCTA, all scaffolded segments were patent. Complete RMS reabsorption was observed in 27 (93%, 95% CI 77–99%) cases. Median minimal lumen diameter (MLD) and area (MLA) of the scaffolded segments were 2.5 [2.1–2.8] mm and 6.4 [4.4–8.4] mm^2^, respectively. Median CT-FFR was 0.88 [0.81–0.91]. Only one (3.5%) vessel showed a flow-limiting CT-FFR value ≤0.80. During the 3-year follow-up, only one (4%) adverse event was observed. **Conclusions:** In patients undergoing RMS implantation, CCTA including noninvasive CT-FFR evaluation is feasible and allows investigation of long-term RMS performance.

## 1. Introduction

**Clinical Trial Registration:**www.clinicaltrials.gov (accessed on 10 September 2022) NCT03327961.

Coronary computed tomography angiography (CCTA) is an established technique for non-invasive evaluation of the coronary lumen and coronary plaques [1]. While its role in the assessment of previously stented coronary vessels is limited due to metal artifacts, previous studies have supported its diagnostic performance in coronary vessels treated with first-generation bioresorbable scaffold (BRS) [2,3,4,5]. Furthermore, anatomical findings can be integrated with non-invasive fractional flow reserve (FFR) derived from CCTA, allowing us to discriminate the presence or absence of flow-limiting disease [6]. After the failure of the first-generation BRS, many alternative platforms have been developed and introduced onto the market, including the second-generation resorbable magnesium scaffold (RMS). Several ongoing sponsored and investigator-initiated studies are investigating the long-term safety and effectiveness of RMS in terms of clinical endpoints, but little evidence has described their performance with CCTA.

The aim of the present investigation is to report clinical outcomes, luminal measurements, and non-invasive FFR assessment using CCTA in the first patients treated with RMS in the Scaffold Implantation in Emilia-Romagna Plus Multi Absorbable Gears Intra Coronary (SHERPA-MAGIC) study.

## 2. Materials and Methods

### 2.1. Study Design

SHERPA-MAGIC is an investigator-driven, multicenter, prospective, single-arm study enrolling patients receiving percutaneous coronary intervention (PCI) with implantation of BRS, in 15 Italian centers. The SHERPA-MAGIC protocol standardizes the optimal indications for patient selection and the BRS implantation technique. The first patient in the SHERPA-MAGIC study was enrolled in December 2017, and recruitment and follow-up remain ongoing. The present analysis considered the first enrolled patients to receive RMS in at least one coronary artery, at the coordinating center of the SHERPA-MAGIC study (University Hospital of Ferrara). As a pre-specified sub-study, these patients underwent CCTA 18 months after index procedure and RMS implantation. The study was conducted in accordance with the ethical principles of the Declaration of Helsinki. Patients were informed that their participation was voluntary, and all gave informed written consent. The study was registered (www.clinicaltrials.gov with identifier NCT03327961) and approved by the ethical review boards at the participating hospitals.

### 2.2. Study Device

Magmaris^®^ (Biotronik AG, Bulach, Switzerland) is a second-generation sirolimus-eluting RMS [7]. The device has a metallic bioresorbable backbone made from magnesium alloy, coated with BIOlute Poly-L-Lactide (PLLA) layer, where the antiproliferative drug is embedded with controlled release for up to 90 days [7]. The device has two tantalum markers, at the proximal and distal ends. The scaffold is placed between the distal and proximal radiopaque marker of the balloon. Previous in vitro and in vivo studies showed that about 95% of the magnesium is reabsorbed within 12 months [7]. The shorter time to resorption is due to a two-step degradation process, in which the magnesium alloy is replaced by a morphous calcium phosphate.

### 2.3. PCI Procedure and RMS Implantation

In the SHERPA-MAGIC study, the decision to proceed with PCI was based on current guidelines and/or institutional protocols, and PCI was performed with standard materials and techniques. The decision to implant RMS was left to the operator and should be consistent with the criteria for patient selection of the SHERPA-MAGIC protocol. The shared criteria qualifying a patient for RMS implantation were: (i) first event (no prior implantation of metallic stent or prior surgical revascularization), (ii) opportunity to pursue complete revascularization in patients with age <65 years, (iii) need for revascularization of long lesions (>24 mm), especially located in left anterior descending (LAD) coronary artery, iv) spontaneous coronary dissection. In addition, the lesion should satisfy the following criteria: (i) reference vessel diameter within 2.8-3.8 mm, (ii) absence of severe coronary calcifications and/or ostial localization and/or bifurcation, considered to suggest a high probability of bifurcation stenting. Patients with clinical indications for oral anticoagulants are not eligible for the study. The SHERPA-MAGIC protocol also standardizes the steps of RMS implantation, which are: (i) mandatory predilatation, (ii) sizing 1:1, (iii) mandatory postdilation with non-compliant balloon less than half of the scaffold diameter. The use of intravascular ultrasonography (IVUS) or optical coherence tomography (OCT) is highly recommended. Standard pharmacotherapy was followed according to the current guidelines, and dual antiplatelet therapy (DAPT) indicated for at least 12 months. The opportunity to continue beyond 12 months is left to the physician and must comply with the indications of guidelines and the internal protocols. Finally, the protocol strongly recommended paying attention to low-density lipoprotein (LDL) targets, aiming to achieve LDL values ≤55 mg/dl as soon as possible.

### 2.4. Coronary Computed Tomography Angiography (CCTA)

CCTA was performed with a Brilliance ICT 256 computed tomography (CT) scanner (Philips, Best, the Netherlands). Standard acquisition techniques were used, which included nitrates before imaging, beta-blockers in patients with a heart rate >65 beats/min, tube setting depending on patient body mass index (80 to 140 kV), and axial scan protocols for patients with lower heart rates in order to reduce radiation doses. CT images were visually graded according to a 3-point scale: 1 (poor), the coronary vessel was not well visualized; 2 (good), the vessel was adequately visualized; and 3 (excellent), the vessel was clearly visualized. All images were transferred to the external workstation for post-processing, analysis, and measurement (Philips Portal Intellispace). CT analyses were performed by two independent reviewers. The radiopaque platinum indicators of the scaffolds were used as landmarks of the scaffolded segment [4]. The mean, minimal, and maximal lumen diameters and areas within the scaffolded segment were determined for each slice [4]. The reference vessel lumen area was calculated as the average of the mean proximal and mean distal vessel areas [4]. The lumen area stenosis was calculated as the reference minus the minimal lumen area as a percentage of the reference lumen area [4]. The scaffolded segments were qualitatively assessed for the presence of noncalcified plaque, calcified plaque, mixed plaque, or high-risk plaque features (positive remodeling, CT attenuation <30 HU, napkin-ring sign, spotty calcium) [8].

### 2.5. CCTA FFR Assessment

Non-invasive CT-FFR analysis was performed blinded in the core laboratory using DeepVessel FFR (DV-FFR) software (Keya Medical, Shenzen, China). DV-FFR is a software medical device designed to extract three-dimensional coronary tree structures and generate noninvasive CT-FFR values from coronary CT angiogram images [6]. It uses deep learning neural networks that encode imaging, structural, and functional characteristics of coronary arteries to learn complex mapping between FFR values and the encoded information [6]. Three-dimensional models of the coronary tree were reconstructed with CCTA only, and the CT-FFR was evaluated for the coronary artery treated with RMS.

### 2.6. Follow-Up and Definitions

Clinical follow-up occurred at one month, six months, and every six months thereafter. For the present analysis, clinical follow-up was censored at three years for each patient. CCTA was performed at the 18-month follow-up. The main endpoints of interest were luminal measurements, morphological characteristics, and CT-FFR values of the target vessels. At CCTA, relevant restenosis was defined as more than 50% of diameter stenosis and/or more than 75% of area stenosis. In terms of CT-FFR, disease was defined as flow-limiting in the presence of values ≤0.80. The clinical endpoint of interest was target vessel failure (TVF), defined as the cumulative occurrence of cardiac death, target vessel myocardial infarction, and ischemia-driven target vessel revascularization. The target vessels were those treated with RMS. Adverse events were defined in agreement with consensus documents [9] and adjudicated by a clinical events committee that reviewed the original source documents.

### 2.7. Statistical Analysis

The present analysis is a proof-of-concept substudy focused on CCTA, and a formal sample size was not required. Based on a previous similar study, our goal was to collect CCTA images from at least 25 patients treated with at least one RMS in at least one coronary vessel [4]. Continuous data were tested for normal distribution with the Kolmogorov–Smirnov test. Normally distributed values were presented as mean ± SD, otherwise, the median value and interquartile range [IQR] were used. Categorical variables were summarized in terms of counts and percentages. All analyses were performed using Stata version 13.1 (StataCorp LP, College Station, TX, USA).

## 3. Results

From December 2017 to July 2018, 34 patients underwent PCI with RMS implantation in the University Hospital of Ferrara. One patient was excluded from the CCTA sub-study due to additional second-generation drug-eluting stent (DES) implantation during the index procedure. Another patient was readmitted to hospital for recurrence of MI not related to the target vessel and underwent new revascularization with DES, 11 months after the index procedure. Additionally, five patients refused to undergo the 18-month CCTA, and for one patient the CCTA quality was poor due to an uncontrolled heart rate at baseline. The demographic data of the 26 patients of the study population are shown in Table 1. Sixteen (61%) patients had single-vessel disease and underwent PCI and RMS implantation. Ten (39%) patients had multi-vessel disease, defined as at least two vessels with stenosis ≥50% as assessed by visual estimation. In two cases, patients underwent PCI with RMS in two and three vessels, respectively, whereas the remaining eight cases were treated only in one vessel, as the others were negative at intracoronary physiology assessment. Overall, 29 coronary arteries were treated with 35 RMS (Table 1). Procedural data and QCA analysis are shown in Table 1. The most frequently involved vessel was the left anterior descendent (LAD) (65%). The median stent length was 25 [20–25] mm, with a median diameter of 3 [3–3.5] mm. High pressure postdilatation using a non-compliant balloon was performed in all cases. No procedural complications were reported. The medical treatment was optimized at discharge and at further clinical visits (Table 1). Within the first six months, all patients achieved low-density lipoprotein (LDL) values <55 mg/dL.

### 3.1. CCTA Findings

**Figure 1:**
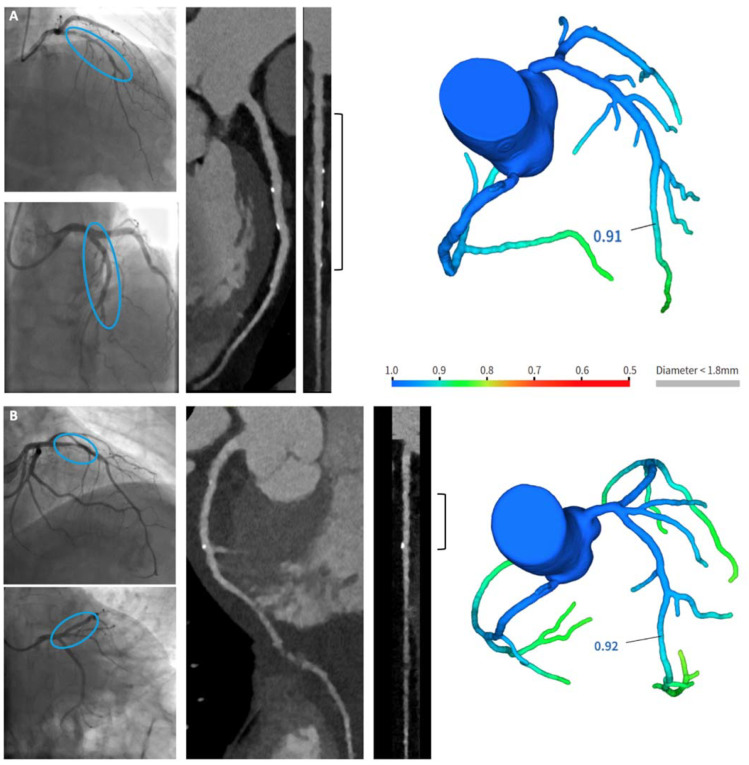

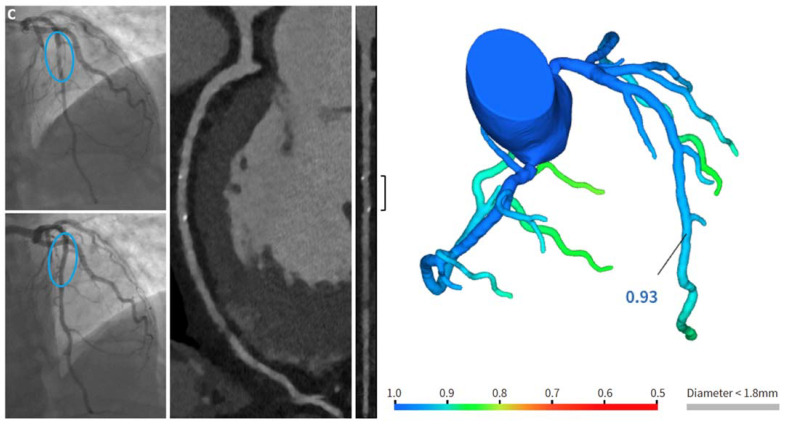
Examples of completely reabsorbed RMS.Each case (**A**–**C**) shows two angiographic images (on the left, from top to bottom: before and after RMS implantation), two CCTA reconstructions (from left to right: curved multiplanar reconstruction and straightened vessel view), and a CT-FFR image. The blue circle in the angiographic images indicates the target segment before and after treatment with RMS. The bracket in the straightened CCTA reconstruction indicates the coronary segment covered with RMS.

**Figure 2:**
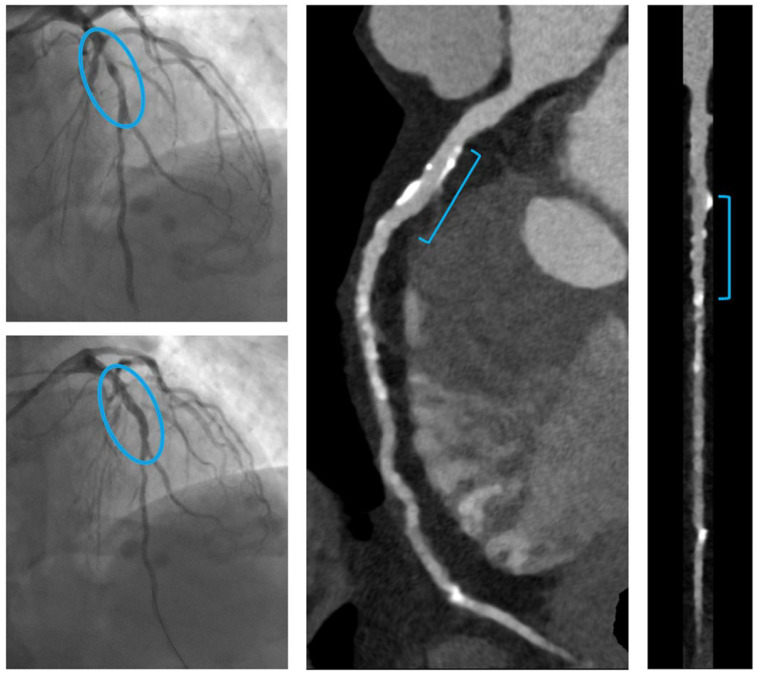
Example of no completely reabsorbed RMS. On the left, from top to bottom, angiographic images show a focal plaque in the middle tract of the LAD, successfully treated by implantation of one RMS (Magmaris^®^ 3x25 mm). Scaffold struts are visible in the CT reconstruction: they appear as bright linear fragments along the treated vessel tract. The colored bar in the FFR-CT image provides a map of FFR-CT values, with blue and green indicating non-significant stenosis.

Quantitative analysis of the scaffolded segment was feasible in all vessels (*n* = 29). In all vessels, the scaffolded segment was easily located through radiopaque edge marker identification. All scaffolded segments were patent. Complete RMS reabsorption was observed in 27 (93%, 95% CI 77–99%) vessels (three exemplificative cases are shown in Figure 1), whereas in two (7%) vessels it was not observed. Figure 2 shows an example of incomplete reabsorption. The median minimal lumen diameter (MLD) and area (MLA) of the scaffolded segments were 2.5 [2.1–2.8] mm and 6.4 [4.4–8.4] mm^2^, respectively. The qualitative plaque analysis of the scaffolded segments revealed that the most common components of residual plaque in the scaffolded segment were fibrous tissue and calcium, suggesting stabilization. In the scaffolded segments, we found no significant hyperplasia proliferation, nor relevant stenoses, nor high-risk plaque features.

A. A patient with long coronary stenosis on the LAD, treated with three RMS (Magmaris^®^ 3.5 × 20 mm, 3.5 × 15 mm, 3 × 25 mm, respectively).
B. A patient with significant stenosis at the bifurcation LAD-first diagonal branch, treated with one RMS on the LAD and balloon on the first diagonal branch (Magmaris^®^ 3 × 25 mm).
C. A patient with a focal culprit plaque on the LAD, treated by one RMS (Magmaris^®^ 2.5 × 25 mm).

The colored bar in the FFR-CT image provides a map of FFR-CT values, with blue and green indicating non-significant stenosis.

### 3.2. Noninvasive CT-FFR Findings

**Figure 3:**
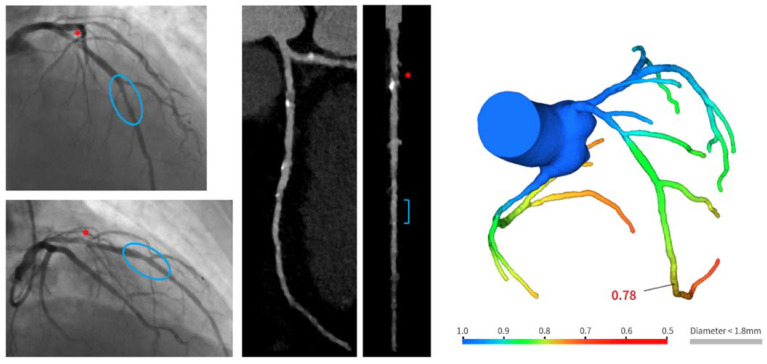
Example of positive CT-FFR on target vessel. Angiographic images show culprit stenosis on the mid LAD (upper image), treated with one RMS (Magmaris^®^ 3 × 25 mm) (lower image). In the angiographic images, the red asterisk indicates an intermediate stenosis in the proximal LAD segment. Follow-up CCTA highlighted the long-term patency of the segment treated with RMS (bracket), showing the progression of the disease in the proximal portion (red asterisk), with the positive presence of prevalent soft plaque at CT-FFR assessment (value 0.78).

Of 29 vessels, noninvasive CT-FFR analysis was feasible in 23 (80%, 95% CI 60–92%). The causes for the missing computation were the presence of severe artifacts (*n* = 4) and the incomplete documentation of the entire coronary artery (*n* = 2). Median CT-FFR in the target vessels was 0.88 [0.81–0.91]. Only one target vessel showed a flow-limiting CT-FFR value (Figure 3) (see detailed description, below).

The colored bar in the FFR-CT image gives a map of FFR-CT values, with blue and green meaning non-significant stenosis, and yellow, orange, and red indicating borderline to significant stenosis.

### 3.3. Year Clinical Outcome

A three-year follow-up was available for all patients. No patient died or experienced reinfarction. One patient received revascularization of the target vessel after the identification of flow-limiting CT-FFR at 18-month CCTA (Figure 3). Although asymptomatic, the presentation involved proximal LAD, and the patient was admitted to hospital to repeat coronary artery angiography. Invasive FFR confirmed the presence of flow-limiting stenosis in the proximal segment of the left anterior descending (LAD) coronary artery, above the scaffolded segment (FFR value 0.76). Revascularization with another RMS implantation was performed. Another patient was admitted to the emergency room for chest pain 12 months after index procedure. Chest pain was atypical, no signs of ischemia were present at the electrocardiogram, and high-sensitivity troponin was negative. No further examination was prescribed.

## 4. Discussion

RMS is a second-generation platform that partially overcomes the major technical drawbacks of first-generation BRS [7]. In the BIOSOLVE II and III trials, RMS showed favorable long-term safety and clinical performance (until five and three years, respectively), with low failure rates for target lesions, and an absence of definite or probable scaffold thrombosis [10,11]. Similar performance has been confirmed in the larger population of the BIOSOLVE-IV study [12], but not in the MAGSTEMI trial [13]. The discrepancy in the available data strongly supports further investigation aiming to clarify and better explain how RMS reabsorption and coronary atherosclerosis stabilization happen in patients receiving RMS implantation. Data from real-life populations with higher anatomical complexity, where RMS implantation is standardized and intensive secondary prevention cardiovascular therapy is applied, are clearly needed to understand the potential benefits of RMS. In the present analysis, we have contributed to generating this evidence by applying the best available non-invasive tool for the assessment of coronary arteries (CCTA plus non-invasive FFR) in subjects participating in an investigator-initiated study that strictly regulated patient selection and implantation techniques. In a preliminary report [14], Salinas and colleagues investigated eight patients with single vessels treated with RMS (one RMS per vessel), including the feasibility of CCTA for follow-up. The authors found that CCTA was feasible, and correctly located and evaluated the patency of RMS [14]. The current paper confirms and extends this data. We analyzed 29 coronary vessels with high clinical and anatomical complexity, and in addition we performed non-invasive FFR computation. We found that scaffolded segments were clearly identified, and quantitative measurements were feasible, as was the computation of noninvasive FFR. 

The second finding refers to the complete reabsorption of RMS after 18 months. Reabsorption was complete in 27 (93%, 95% CI 77–99%) scaffolded segments, and no significant impact was observed in terms of anatomical or functional data. In addition, qualitative analysis of scaffolded segments revealed a tendency towards plaque stabilization with a dominant presence of fibrous tissue and calcium, and a lack of high-risk plaque features. This latter point deserves particular attention because the clinical goal in the management of CV patients is the stabilization and possible regression of the atherosclerotic process. This goal cannot be achieved by the implantation of metallic platforms, but only by aggressive lipid lowering and anti-remodeling treatments. In the same direction, the development of anti-inflammatory drugs aims to reduce the inflammatory burden on atherosclerotic plaques and to reduce their recurrence [15,16]. Finally, it is important to note that most of the patients in the present analysis were admitted to hospital for MI (82%, 95% CI 63–94%). It could be speculated that the MAGSTEMI trial revealed negative outcomes due to the enrollment of STEMI patients, compared to a prevalent stable population in the BIOSOLVE studies [10,11,12,13]. Although speculative due to the small sample size, our findings seem to suggest that patient selection and implantation techniques are more important than clinical presentation in determining long-term outcomes after RMS implantation.

## 5. Limitations

Several limitations should be acknowledged concerning the present analysis. First, the study population was small and does not allow definitive conclusions to be drawn, particularly in terms of clinical outcomes. The SHERPA-MAGIC study and other similar studies to investigate the clinical benefit of RMS are ongoing. The implantation technique was strictly standardized, and it is not clear whether similar findings can be achieved with different approaches. Similarly, enrolled patients received strict follow-up and aggressive secondary CV prevention, with particular attention to low-density lipoprotein targets. We cannot exclude the possibility that this affected the observed good performance. CCTA was performed just once, at 18 months, and we did not have baseline images to allow comparison and address potential variations. However, this was beyond the study’s scope, which aimed to demonstrate the feasibility and reliability of CCTA and noninvasive FFR. Finally, plaque composition in the scaffolded segments was assessed by qualitative analysis and not by the application of dedicated software. The functional role of the scaffolded segments was assessed by non-invasive FFR.

## 6. Conclusions

In a highly selective study population of patients undergoing RMS implantation using a standardized technique, we confirmed that CCTA is feasible and allows discrimination of long-term anatomical and functional performance of scaffolded segments.

## Figures and Tables

**Table 1:** Baseline characteristics of the population.

	Total
	Patients (*n* = 26)
Age (years)	59 ± 7
Male sex (%)	22 (85)
**CV risk factors (%)**	
Diabetes	1 (3.8)
Hypertension	13 (50)
Hyperlipidemia	14 (54)
Current smoker	11 (42)
**Medical history (%)**	
MI and/or coronary revascularization	0 (0)
PAD	1 (4)
CKD	3 (11)
**Clinical presentation**	
STEMI (%)	8 (31)
NSTEMI (%)	15 (58)
CCS (%)	3 (11)
**Medical therapy (%)**	
Aspirin	26 (100)
P2Y12 inhibitor	26 (100)
ACE inhibitor/A2R blocker	23 (88)
High-potency statin	24 (92)
Ezetimibe	18 (69)
PCSK9 inhibitor	4 (15)
	**Vessels (*n* = 29)**
Target vessel (%)	
-left anterior descending	19 (66)
-left circumflex	5 (17)
-right coronary	5 (17)
AHA/ACC classification (%)	
-A, B1	6 (21)
-B2	15 (52)
-C	8 (27)
Reference vessel diameter, mm	2.7 (2.5–3.3)
Minimal lumen diameter, mm	0.6 (0.3–0.8)
Diameter stenosis, %	77 (66–95)
Lesion length, mm	18 (15–21)
Predilatation (%)	28 (100)
Largest predilatation balloon, mm	2.5 (2.5–3)
RMS diameter, mm	3 (3–3.5)
Total RMS length, mm	25 (20–25)
Overlapping RMS (%)	6 (21)
Postdilatation (%)	28 (100)
Largest postdilatation balloon, mm	3.5 (3.5–3.75)
Intracoronary imaging, no. (%)	20 (69)

CV: cardiovascular. MI: myocardial infarction. PAD: peripheral artery disease. CVA: cerebrovascular accident. CKD: chronic kidney disease, defined as CrCl < 60 mL/min. COPD: chronic obstructive pulmonary disease. STEMI: ST-segment elevation MI. NSTEMI: no ST-segment elevation MI. CCS: chronic coronary syndrome. ACE: angiotensin converting enzyme. A2R: angiotensin 2 receptor. AHA: American Heart Association. ACC: American College of Cardiology. RMS: resorbable magnesium scaffold.
